# Aspirin prevents estrogen deficiency-induced bone loss by inhibiting osteoclastogenesis and promoting osteogenesis

**DOI:** 10.1186/s13018-023-03710-y

**Published:** 2023-03-22

**Authors:** Yongyun Chang, Keyu Kong, Zhicheng Tong, Hua Qiao, Yi Hu, Runzhi Xia, Jingwei Zhang, Zanjing Zhai, Huiwu Li

**Affiliations:** Shanghai Key Laboratory of Orthopaedic Implants, Department of Orthopaedics, Ninth People’s Hospital, Shanghai Jiao Tong University School of Medicine, No.639, Zhizaoju Road, Shanghai, China

**Keywords:** Aspirin, Osteoclastogenesis, Osteogenesis, Osteoporosis, NF-*κ*B, MAPK

## Abstract

**Background:**

Aspirin is a commonly used antipyretic, analgesic, and anti-inflammatory drug. Numerous researches have demonstrated that aspirin exerts multiple biological effects on bone metabolism. However, its spatiotemporal roles remain controversial according to the specific therapeutic doses used for different clinical conditions, and the detailed mechanisms have not been fully elucidated. Hence, in the present study, we aimed to identify the dual effects of different aspirin dosages on osteoclastic activity and osteoblastic bone formation in vitro and in vivo.

**Methods:**

The effects of varying doses of aspirin on osteoclast and osteoblast differentiation were evaluated in vitro. The underlying molecular mechanisms were detected using quantitative real-time polymerase chain reaction, western blotting, and immunofluorescence techniques. An ovariectomized rat osteoporosis model was used to assess the bone-protective effects of aspirin in vivo.

**Results:**

Aspirin dose-dependently suppressed RANKL-induced osteoclasts differentiation and bone resorption in vitro and reduced the expression of osteoclastic marker genes, including TRAP, cathepsin K, and CTR. Further molecular analysis revealed that aspirin impaired the RANKL-induced NF-*κ*B and MAPK signaling pathways and prevented the nuclear translocation of the NF-*κ*B p65 subunit. Low-dose aspirin promoted osteogenic differentiation, whereas these effects were attenuated when high-dose aspirin was administered. Both low and high doses of aspirin prevented bone loss in an ovariectomized rat osteoporosis model in vivo.

**Conclusion:**

Aspirin inhibits RANKL-induced osteoclastogenesis and promotes osteogenesis in a dual regulatory manner, thus preventing bone loss in vivo. These data indicate that aspirin has potential applications in the prevention and treatment of osteopenia.

## Introduction

Bone tissue is a rigid and dynamic tissue that undergoes continuous remodeling and repair. Bone homeostasis requires a balance between osteoblastic bone formation and osteoclastic bone resorption [1]. The osteoclast is a giant multinucleated cell that absorbs bone matrix from the hematopoietic lineage [2]. Osteoclast differentiation and activation are dependent on two essential cytokines: macrophage colony-stimulating factor (M-CSF) and receptor activator of nuclear factor-(*κ*B) (RANK) ligand [3]. The binding of RANKL and the RANK receptor is crucial for the induction of multiple downstream targets, including the NF-*κ*B and MAPK pathways, resulting in the activation of NFATc1 and c-Fos [4]. Osteoblasts, the bone-forming cells of the remodeling unit, are responsible for the deposition of the new bone matrix and mineralization [5]. Excessive activity of osteoclasts or osteoblast dysfunction can cause an imbalance in bone remodeling and thus induce osteopenia diseases, such as osteoporosis [6]. Therefore, using agents with both anabolic and antiresorptive functions to synchronously target osteoclasts and osteoblasts may be an optimal strategy for osteoporosis prevention and treatment.

Aspirin, also known as acetylsalicylic acid (ASA), is widely used for its antipyretic, analgesic, and anti-inflammatory activities by inhibiting cyclooxygenase [7]. It is also recommended for the primary and secondary prevention of cardiovascular diseases, such as angina pectoris, acute myocardial infarction, transient ischemic attack, and peripheral vascular disease at low doses with antiplatelet effects [8]. Previous epidemiological and fundamental studies have demonstrated that aspirin exerts multiple biological effects on bone metabolism, however, its dose-dependent roles remain controversial, and the detailed mechanisms have not been fully elucidated [9]. Epidemiological studies demonstrated that daily use of low-dose aspirin may inhibit bone loss and preserve bone mineral density (BMD) [10, 11], whereas, other clinical studies showed that the chronic use of low-dose aspirin was not associated with lower BMD in the general population [12, 13]. Numerous studies have demonstrated that aspirin mediates the osteoclasts and osteoblasts located on the bone remodeling area [14, 15]. These cells orchestrate a complex metabolic scenario, resulting in degradative or synthetic functions for bone mineral tissues. However, evidence of the relationship between aspirin and bone homeostasis remains inconclusive according to the specific therapeutic doses used for different clinical conditions.

In the present study, we aimed to investigate the different roles of aspirin in osteoblastic bone formation and osteoclastic bone resorption in a dose-dependent manner. An ovariectomized (OVX)-induced rat osteoporosis model was established to examine the protective role of aspirin against bone loss in vivo. Our findings suggest that aspirin is a promising approach for the prevention of osteoporosis.

## Methods

### Reagents

Alpha minimum essential medium (MEM), fetal bovine serum (FBS), and penicillin–streptomycin solution were purchased from Gibco (Gaithersburg, MD, USA). Tartrate-resistant acid phosphatase (TRAP) staining solution and alkaline phosphatase (ALP) staining kit were obtained from Sigma-Aldrich (St. Louis, MO, USA). Alizarin red S (ARS) solution was purchased from Cyagen (Santa Clara, CA, USA). Cell Counting Kit-8 (CCK-8) was obtained from Dojindo Molecular Technology (Dojindo, Kumamoto, Japan). Recombinant mouse M-CSF and RANKL were obtained from R&D Systems (Minneapolis, MN, USA). Primary antibodies against p-ERK1/2 (9101, 1:1000), ERK1/2 (9102, 1:1000), p-JNK (4668, 1:1000), JNK (9252, 1:1000), p-p38 (4511, 1:1000), p38 (8690, 1:1000), p-p65 (3033, 1:1000), p65 (8242, 1:1000), I*κ*B*α* (4814, 1:1000), NFATc1 (8032, 1:1000), c-Fos (2250, 1:1000), TRAP (15,094, 1:1000), and GAPDH (5174, 1:1000) were obtained from Cell Signaling Technology (Danvers, MA). Primary antibodies against OCN (ab93876, 1:100) were obtained from Abcam (Cambridge, MA).

### In vitro* osteoclastogenesis assay*

Bone marrow-derived macrophages (BMMs) were extracted from the femur of C57/BL6 mice and cultured in a complete growth medium containing M-CSF (30 ng/ml). Cells were seeded in a 96-well plate at a density of 1 × 10^4^ cells/well and treated with M-CSF (30 ng/ml) and RANKL (50 ng/ml) in the presence or absence of various concentrations of aspirin (10, 50, 100, 150, and 200 μg/ml). After osteoclast formation, the cells were fixed with 4% paraformaldehyde for 20 min and stained for TRAP activity. TRAP-positive multinucleated cells with three or more nuclei were counted as osteoclasts.

### Resorption pit assay

BMMs were cultured in hydroxyapatite-coated 96-well plates (Corning, NY, USA) at a density of 1 × 10^4^ cells/well and treated with M-CSF (30 ng/ml), RANKL (50 ng/ml), and different concentrations of aspirin (0, 10, 50, 100, 150, and 200 μg/ml). After osteoclasts were formed, a 10% sodium hypochlorite solution was used to remove the cells so that the resorption area could be observed with standard light microscopy and then quantified using the ImageJ software (NIH, Bethesda, MD, USA).

### Actin ring formation assay

BMMs were plated into 96‐well plates at a density of 1 × 10^4^ cells/well and treated with increasing concentrations of aspirin (0, 10, 50, 100, 150, and 200 μg/ml) in the presence of M-CSF (30 ng/ml) and RANKL (50 ng/ml) for 5 days. The 4% paraformaldehyde was used to fix cells for 20 min at room temperature. The cells were permeabilized with 0.5% Triton X‐100 and blocked with 3% bovine serum albumin (BSA) in PBS. The F-actin rings were then stained with rhodamine-conjugated phalloidin (Cytoskeleton, Denver, USA) and counterstained with DAPI. Images were captured using a fluorescence microscope (Olympus). The number of multinucleated cells with three or more nuclei was counted.

### Cytotoxicity assay

BMMs were seeded into 96-well plates at a density of 1 × 10^4^ cells/well and incubated overnight. The cells were treated with various concentrations of aspirin (0, 10, 50, 100, 150, and 200 μg/ml) for 48 h. At the end of the experimental period, the cells were incubated with 10 μl of CCK-8 reagent and 100 μl of culture medium for another 2 h at 37 °C. The optical density values were measured using a spectrophotometer at 450 nm on an Infinite M200 Pro multimode microplate reader (Tecan Life Sciences, Switzerland).

### Quantitative real-time polymerase chain reaction (PCR) analysis

BMMs were seeded into 6-well plates at the density of 3 × 10^5^ cells/well and treated with different concentrations of aspirin (0, 10, 50, 100, 150, and 200 μg/ml) in the presence of M-CSF (30 ng/ml) and RANKL (50 ng/ml) for 5 days. Total RNA was extracted with TRIzol reagent (Invitrogen, Carlsbad, CA, USA), and cDNA was synthesized by a reverse transcriptase master kit (TaKaRa Biotechnology, Osaka, Japan). Quantitative real-time PCR was performed with the SYBR Green PCR kit (TaKaRa Biotechnology, Osaka, Japan). Primer sequences used are listed in Table 1. The expression levels of the target genes were normalized to GAPDH. Results were calculated using the 2^−ΔΔ*Ct*^ method.

**Table 1:** Sense and anti-sense primers for quantitative real-time PCR

Genes	Forward (5′–3′)	Reverse (5′–3′)
TRAP	CTGGAGTGCACGATGCCAGCGACA	TCCGTGCTCGGCGATGGACCAGA
Cathepsin K	CTTCCAATACGTGCAGCAGA	TCTTCAGGGCTTTCTCGTTC
CTR	TGCAGACAACTCTTGGTTGG	TCGGTTTCTTCTCCTCTGGA
NFATc1	CCGTTGCTTCCAGAAAATAACA	TGTGGGATGTGAACTCGGAA
MMP9	CGTGTCTGGAGATTCGACTTGA	GGAAACTCACACGCCAGA
GAPDH	AGGTCGGTGTGAACGGATTTG	TGTAGACCATGTAGTTGAGGTCA

### Western blotting analysis

BMMs were incubated in 6-well plates at a density of 3 × 10^5^ cells/well and treated with RANKL (50 ng/ml) in the presence or absence of aspirin for the indicated time. The cells were rinsed three times with pre-cooled PBS and lysed with RIPA buffer containing 1% protease and phosphatase inhibitors for 30 min on ice. The cell lysate was centrifuged at 12,000 g for 15 min to collect the protein. A bicinchoninic acid assay kit was used to measure protein concentrations. Proteins were separated by SDS-PAGE and transferred onto nitrocellulose membranes. Non-specific binding was blocked with 5% BSA for 1 h at room temperature. The membranes were then incubated with primary antibodies at 4 °C overnight. The membranes were then incubated with secondary antibodies for 1 h at room temperature. Images were captured with an Odyssey fluorescent imaging system (LI-COR Biosciences, Lincoln, NE, USA).

### NF-κB p65 subunit localization assay

BMMs were seeded onto 12-well plates containing glass coverslips at a density of 1 × 10^5^ cells/well and cultured for 24 h. The cells were treated with or without aspirin for 2 h, followed by stimulation with RANKL (50 ng/ml) for 30 min. The cells were washed with PBS and fixed with 4% paraformaldehyde for 30 min. Cells were permeabilized with 0.5% Triton X‑100 for 30 min and blocked with 3% BSA for 30 min. The anti-NF-*κ*B p65 subunit antibody was incubated overnight at 4 °C, and the nuclei were counterstained with DAPI for 10 min. Images of p65 translocation were acquired using a laser-scanning confocal microscope (Leica, Wetzlar, Germany).

### ALP and ARS staining assay

MC3T3-E1 cells were seeded into 24-well plates at a density of 5 × 10^4^ cells/well and cultured in an osteogenic induction medium containing 100 nM dexamethasone, 10 mM *β*-glycerophosphate, and 50 μM ascorbic acid. Increasing concentrations of aspirin (0, 10, 50, 100, 150, and 200 μg/ml) with or without TNF-*α* (10 ng/ml) were added to the osteogenic induction medium to induce cells for 7 or 21 days. On day 7, the ALP activity was measured using an ALP staining kit. On day 21, the mineralization of cells was assessed using ARS staining. Briefly, cells were washed twice with PBS and fixed with 4% paraformaldehyde for 30 min. The ALP staining kit and ARS solution were used according to the manufacturer’s instructions.

### OVX-induced osteoporosis rat model

Animal experiments were conducted with permission from the Animal Care and Experimentation Ethics Committee of Shanghai Ninth People’s Hospital, Shanghai Jiao Tong University School of Medicine. Twenty-four female Sprague–Dawley rats aged 8 weeks were randomly divided into four groups (*n* = 6): (1) sham, (2) OVX, (3) OVX + low-dose aspirin (6 mg/kg/day), and (4) OVX + high-dose aspirin (30 mg/kg/day). Bilateral ovariectomy was performed to induce osteoporosis in the OVX, OVX + low-dose aspirin, and OVX + high-dose aspirin groups. A sham procedure without ovarian resection was performed in the sham group. The animals were raised in a standard environment with a normal diet. One week after surgery, aspirin was administered orally once a day for 12 consecutive weeks in the OVX + low-dose aspirin and OVX + high-dose aspirin groups. The rats in the sham and OVX groups were administered an equivalent volume of normal saline. After that, all animals were sacrificed, and tibia samples were collected for micro-CT and histological analysis.

### Micro-CT scanning and analysis

Samples without excess muscle tissue were fixed in 4% paraformaldehyde and then scanned with a high-resolution micro-CT scanner (Skyscan, Aartselaar, Belgium) with the following settings: X-ray voltage, 50 kV; electric current, 500 µA. Finally, three-dimensional (3D) images were reconstructed, and microstructure indices were analyzed, including bone volume/total tissue volume (BV/TV), trabecular number (Tb.N), trabecular thickness (Tb.Th), and trabecular space (Tb.Sp).

### Histological analysis

Following micro-CT scanning, the retrieved tibia specimens were soaked in a 10% EDTA decalcifying solution. After decalcification, the samples were embedded in paraffin and sectioned into 5-µm-thick slides. TRAP staining and immunohistochemical staining using an anti-OCN primary antibody were performed for histological examination. The number of osteoclasts per bone surface (N.Oc/BS) and the number of OCN-positive cells were calculated using the ImageJ software.

### Statistical analysis

Data obtained from at least three duplicate experiments are presented as mean ± standard deviation. Differences between three or more groups were evaluated by one-way analysis of variance followed by the Student–Newman–Keuls post hoc test, and differences between two groups were analyzed by student’s* t* test. The statistical significance level was set at *p* < 0.05.

## Results

### *Aspirin inhibited RANKL-induced osteoclastogenesis *in vitro

**Fig. 1:**
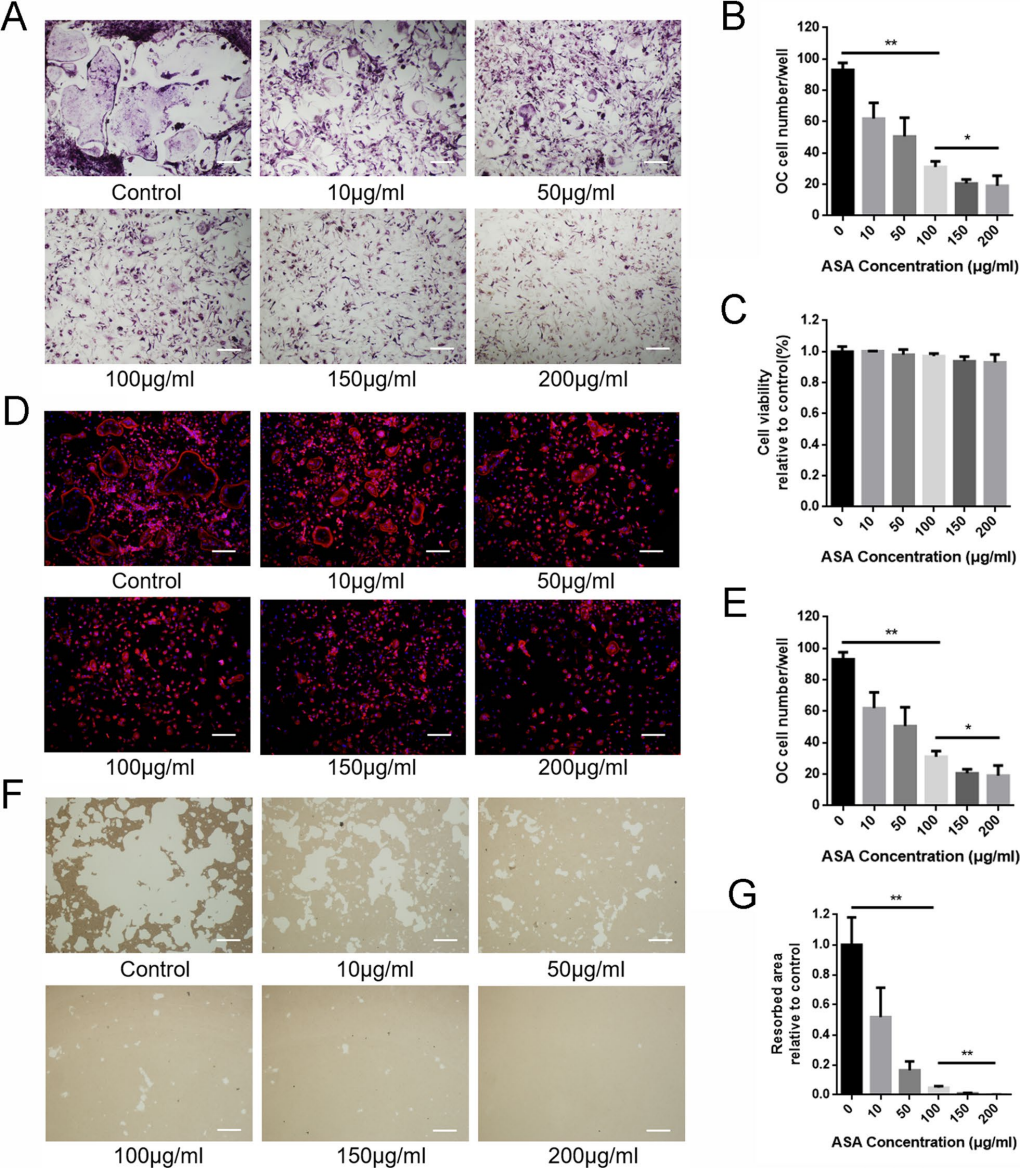
Aspirin inhibited RANKL-induced osteoclastogenesis in a dose-dependent manner without cytotoxicity. **A** Representative images of TRAP staining treated with increasing concentrations of aspirin. **B** Quantitative analysis of the number of TRAP-positive cells with more than 3 nuclei. **C** The cytotoxicity of aspirin was evaluated by CCK-8 assay. **D** Representative images of fluorescence staining treated with various concentrations of aspirin. **E** Quantitative analysis of the number of osteoclasts. **F** Representative images of hydroxyapatite resorption treated with different concentrations of aspirin. **G** Quantitative analysis of the area of hydroxyapatite resorption. Scale bar = 10 μm. *P < 0.05, **P < 0.01

To determine the effects of aspirin on osteoclastogenesis, BMMs were treated with various concentrations of aspirin as previously described. According to TRAP staining results, aspirin suppressed RANKL-induced osteoclast formation. Moreover, increasing concentrations of aspirin had a dose-dependent effect on osteoclastogenesis (Fig. 1A, B). Cell viability assays were performed to investigate aspirin’s potential cytotoxicity in BMMs. The CCK-8 assay results showed that aspirin had no significant inhibitory effect on BMM proliferation at the concentrations used in this study, even at high concentrations (Fig. 1C). Altogether, these data revealed that aspirin inhibited osteoclast formation in a concentration-dependent manner without any obvious cytotoxic effects.

### *Aspirin impaired RANKL-induced F-actin ring formation and osteoclastic bone resorption *in vitro

Podosomes are essential for osteoclastic mobility and bone resorption functions [16]. Hence, we performed rhodamine-conjugated phalloidin staining to visualize osteoclast podosomes. The images showed that osteoclasts with large intact F-actin rings and multiple nuclei were observed in the control group, whereas smaller and fewer osteoclasts with fewer nuclei were detected under different concentrations of aspirin treatment (Fig. 1D, E).

A hydroxyapatite resorption assay was performed to investigate whether aspirin could impair the bone-resorptive function of osteoclasts. As shown in Fig. 1F and G, aspirin significantly reduced the resorption area in a concentration-dependent manner. Osteoclastic bone resorption was almost completely suppressed by 200 μg/ml aspirin treatment. Our results indicated that aspirin impaired the bone resorption activity of osteoclasts.

### Aspirin suppressed the expression of osteoclastic-specific genes

**Fig. 2:**
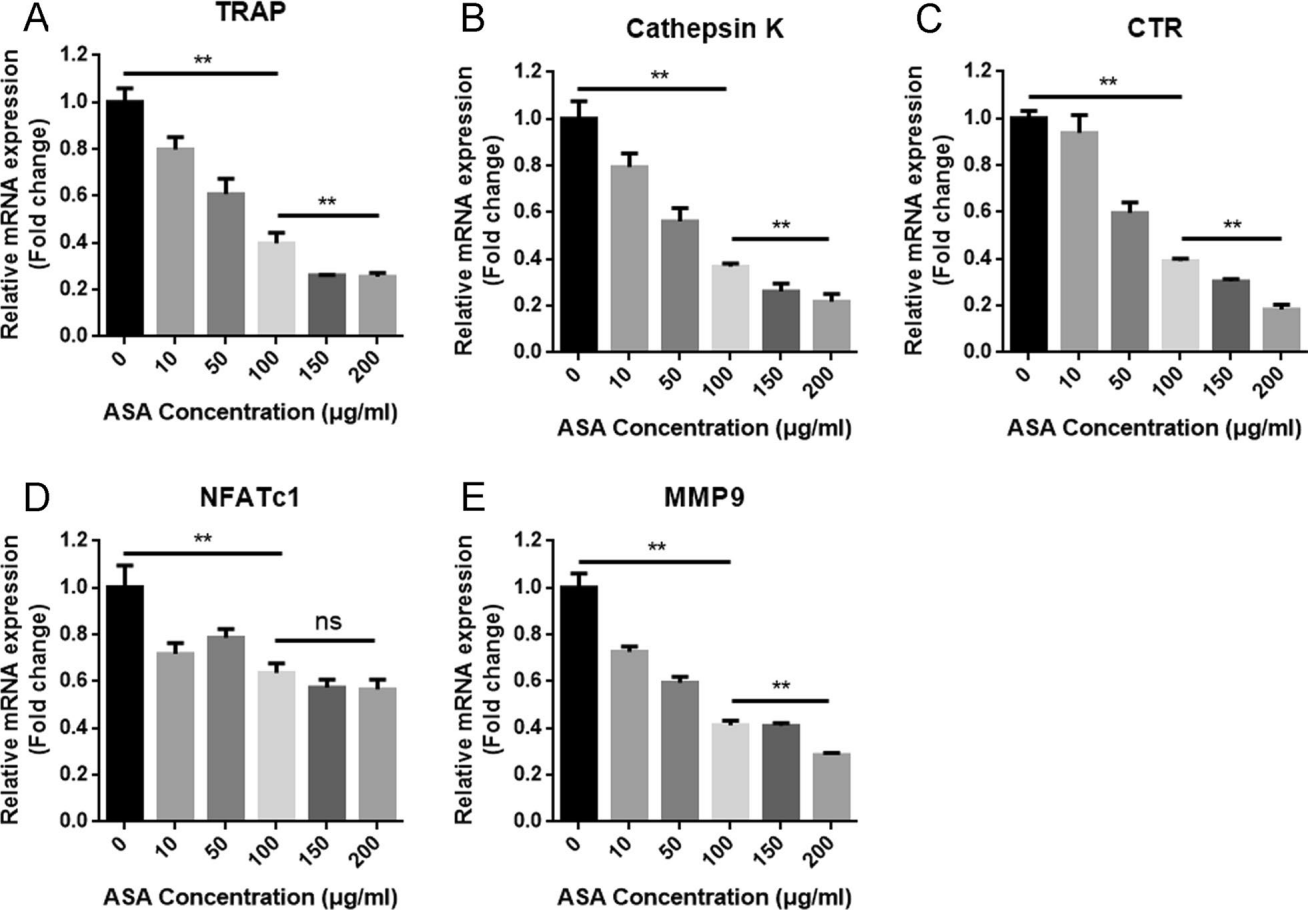
Aspirin suppressed the expression of osteoclastic-specific genes. **A** TRAP, **B** Cathepsin K, **C** CTR, **D** NFATc1, **E** MMP9. GAPDH was used as an internal control. **P < 0.01

The effect of aspirin on RANKL-induced osteoclastic gene expression was examined using quantitative real-time PCR. The results demonstrated that aspirin treatment significantly suppressed the expression of osteoclastic marker genes, including TRAP, cathepsin K (CTSK), calcitonin receptor (CTR), nuclear factor of activated T-cells cytoplasmic 1 (NFATc1), and matrix metalloproteinase-9 (MMP9) (Fig. 2A–E). Therefore, these results further confirmed that aspirin suppressed the expression of RANKL-induced osteoclast-specific genes in vitro.

### Aspirin attenuated RANKL-induced NF-κB and MAPK pathways activation

**Fig. 3:**
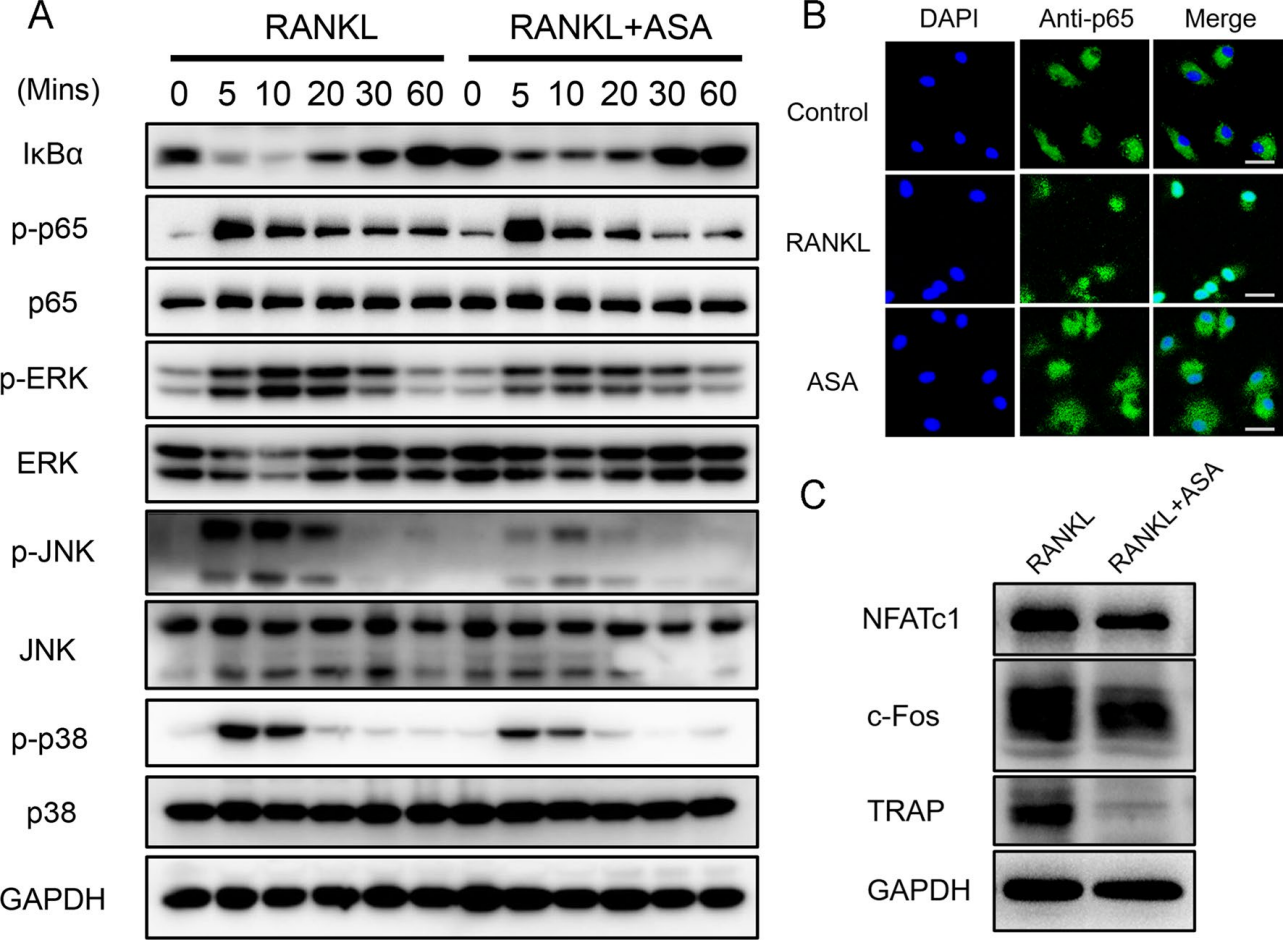
Aspirin attenuated RANKL-induced NF-*κ*B and MAPK signaling pathways activation. **A** The NF-*κ*B and MAPK protein levels were examined with western blotting. **B** The localization of NF-*κ*B p65 subunit was represented with immunofluorescence assay. Scale bar = 20 μm. **C** The effect of aspirin on the expression of osteoclastic protein, including NFATc1, c-Fos and TRAP. GAPDH was used as an internal control

To further understand the mechanism underlying the effects of aspirin on osteoclastogenesis, the RANKL-induced downstream signaling pathways were explored. The binding of RANKL to RANK induces the recruitment of TRAF6, thereby initiating the phosphorylation and degradation of inhibitor *κ*B*α* (I*κ*B*α*), resulting in activation of the NF-*κ*B pathway [17]. Our results showed that aspirin treatment markedly attenuated RANKL-mediated degradation of I*κ*B*α* (Fig. 3A). Similarly, aspirin treatment inhibited the phosphorylation of the p65 subunit of NF-*κ*B (Fig. 3A).

Activation of the MAPK signaling pathway plays a pivotal role in osteoclast formation and differentiation [18]. To evaluate the effect of aspirin on the MAPK pathway, we characterized the phosphorylation of ERK1/2, JNK, and p38, which are the three major subfamilies of the MAPK pathway. The results showed that the phosphorylation of ERK1/2, JNK, and p38 was significantly attenuated by aspirin treatment (Fig. 3A).

NF-*κ*B subunits are sequestered by binding to I*κ*B*α* in the cytoplasm. The phosphorylation and degradation of I*κ*B*α* liberate the p65 subunit of NF-*κ*B to enter the cell nucleus and bind to target sites. Immunofluorescence staining demonstrated that the p65 subunit was notably translocated into the nucleus induced by RANKL treatment compared to the control group. However, aspirin treatment reduced this translocation (Fig. 3B).

NFATc1 is a crucial transcription factor involved in RANKL-induced osteoclast differentiation [19]. Our results showed that the expression of NFATc1 was significantly inhibited after treatment with aspirin for 5 days (Fig. 3C). Moreover, aspirin treatment reduced the expression of osteoclastogenesis-associated proteins, such as c-Fos and TRAP (Fig. 3C). Overall, our results indicated that aspirin treatment attenuated RANKL-induced NF-*κ*B and MAPK pathway activation.

### *Aspirin promoted osteogenic differentiation *in vitro

**Fig. 4:**
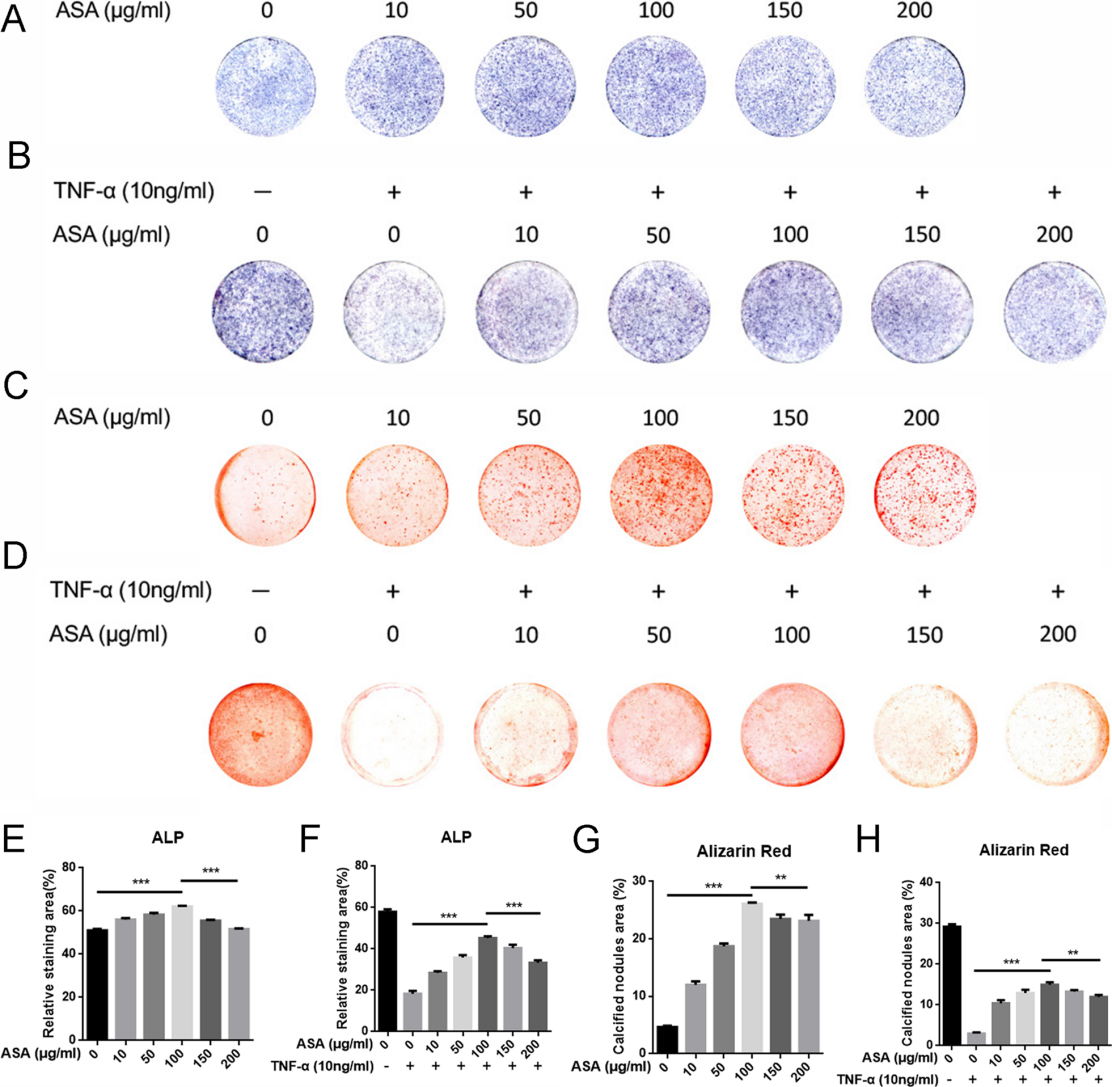
Aspirin promoted osteogenic differentiation in vitro. **A** Representative images of ALP staining treated with various concentrations of aspirin. **B** Aspirin treatment rescued the impaired ALP activity induced by TNF-*α*. **C** Representative images of Alizarin Red staining treated with different concentrations of aspirin. **D** Aspirin treatment reserved the decreased calcified deposits induced by TNF-*α*. **E**–**H** Quantitative analyses of ALP and Alizarin Red staining. **P < 0.01, ***P < 0.001

We next examined the effects of aspirin treatment on osteogenic differentiation potential. The results of ALP and ARS staining showed that low concentrations of aspirin (10, 50, and 100 μg/ml) gradually promoted osteogenic differentiation, while these facilitating effects were attenuated when high concentrations of aspirin (150, and 200 μg/ml) were administered (Fig. 4A, C). At a concentration of 100 μg/ml, aspirin showed a maximal increase in osteogenic differentiation capacity.

TNF-*α* impairs osteogenic differentiation. Since aspirin has been reported to inhibit the function of TNF-*α* [20], we investigated the effect of aspirin treatment on TNF-*α*-induced osteogenesis impairment. TNF-*α* notably suppressed the osteogenic differentiation of osteoblasts, as evidenced by the reduced ALP activity and mineral nodule formation (Fig. 4B, D). Aspirin treatment significantly rescued the ALP activity and calcified deposits under inflammatory conditions (Fig. 4B, D). In addition, aspirin at a dose of 100 μg/ml exerted the most efficient reversal effects (Fig. 4B, D). The results of the quantitative analysis are shown in Figs. 4E–H.

### Aspirin prevented OVX-induced bone loss

**Fig. 5:**
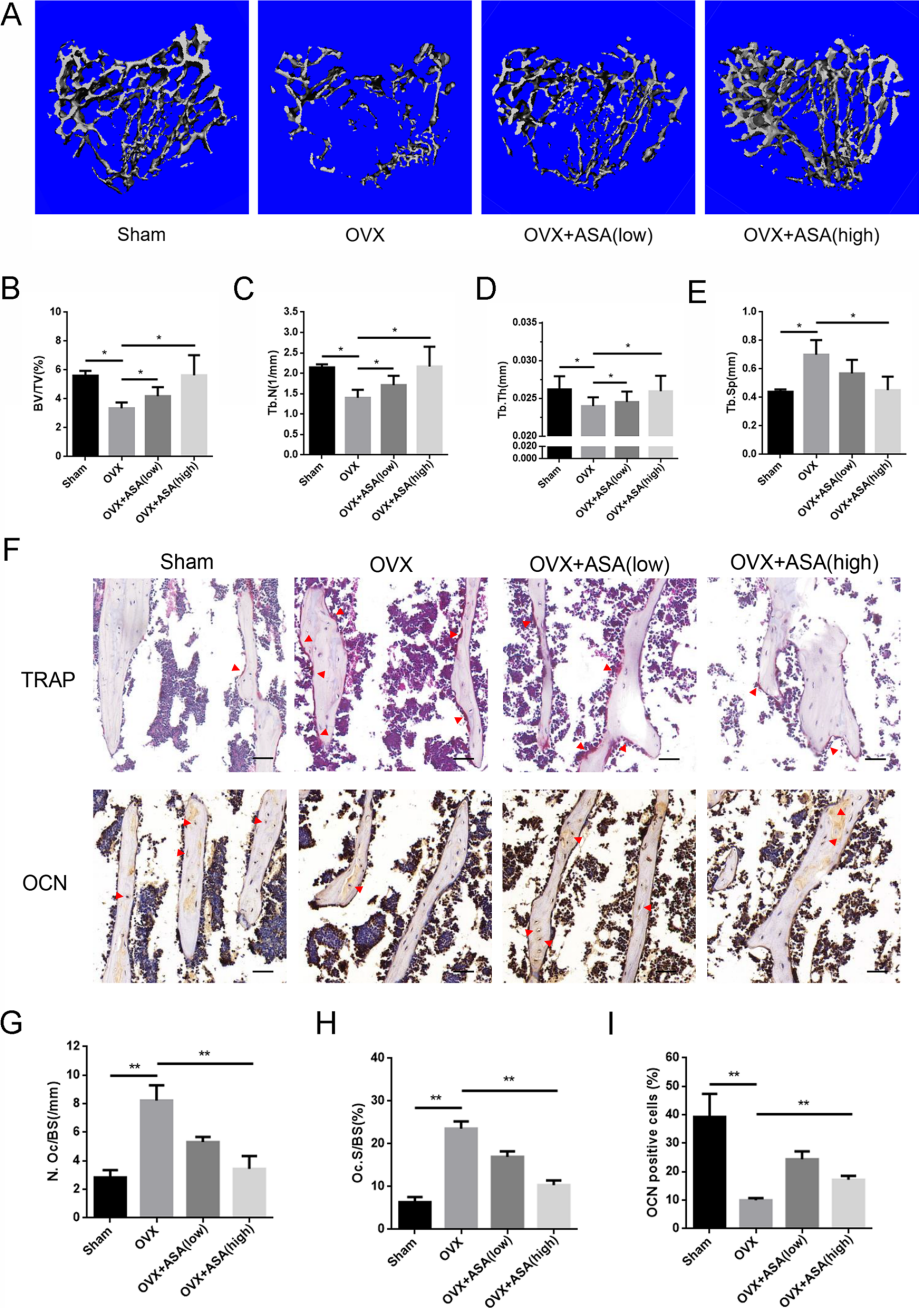
Aspirin prevented OVX-induced bone loss in vivo. **A** Representative images of Micro-CT in sham, OVX and aspirin treatment (low and high doses) groups. **B**–**E** Quantitative analyses of bone microstructure parameters, including BV/TV, Tb.N, Tb.Th and Tb.Sp. **F** Representative images of TRAP and OCN immunohistochemical staining in sham, OVX and different doses of aspirin treatment groups. Scale bar = 50 μm. **G**–**I** Quantitative analyses of osteoclast number/bone surface (N.Oc/BS), osteoclast surface/bone surface (Oc.S/BS) and OCN positive cells ratios. *P < 0.05, **P < 0.01

To further assess the potential bone-protective effects of aspirin in vivo, we employed an ovariectomized rat osteoporosis model to mimic postmenopausal osteoporosis. In our study, the dose of aspirin (6 mg/kg/day) used in the OVX + low-dose aspirin group was equivalent to that (100 mg/day) prescribed to prevent human cardiovascular events in clinical practice. Micro-CT revealed a markedly decreased trabecular bone mass in the OVX group compared with that in the sham group (Fig. 5A). However, treatment with aspirin (6 or 30 mg/kg/d) prevented the extent of bone loss induced by OVX (Fig. 5A), with significant increases in BV/TV, Tb.N, and Tb. Th and a decrease in Tb.Sp (Fig. 5B–E).

A histological examination was performed to analyze the effect of aspirin on OVX-induced bone loss. The results demonstrated that aspirin treatment (6 or 30 mg/kg/day) significantly decreased the number of TRAP-positive cells compared to that in the OVX group (Fig. 5F–H). In contrast, the results of osteocalcin (OCN) immunohistochemical staining showed that more OCN-positive cells were found in the aspirin treatment (6 or 30 mg/kg/day) groups than in the OVX group (Fig. 5F, I). In conclusion, our data indicate that aspirin can prevent OVX-induced bone loss in vivo.

## Discussion

Aspirin is one of the oldest medications that belongs to the classical nonsteroidal anti-inflammatory drug (NSAID) family. It inhibits cyclooxygenase 1(COX-1) and cyclooxygenase 2(COX-2) enzymes in an irreversible and nonselective manner [21]. High-dose aspirin is generally administered to alleviate pain and inflammatory reactions. Low-dose aspirin is widely recommended for the prevention of cardiovascular and cerebrovascular diseases in patients at a high risk of thrombogenesis [22]. Low-dose aspirin is also an alternative strategy for preventing venous thromboembolism after orthopedic surgery [23, 24]. Beyond the traditional functions mentioned above, increasing evidence demonstrates that regular long-term administration of low-dose aspirin can diminish the incidence and mortality of several types of cancer, including gastrointestinal, breast, and prostate cancer [25, 26]. Aspirin has an emerging potency in many fields with old or new mechanisms.

Osteoporosis is a common systemic skeletal disease characterized by low bone mass and microarchitectural deterioration of the bone tissue, leading to enhanced bone fragility and increased fracture risk [27]. Previous studies have shown that aspirin may be an appropriate intervention for osteoporosis. However, the clinical effects of regular aspirin use on BMD and skeletal regeneration in the elderly population remain conflicting and inconclusive based on previous epidemiological studies. According to a study by Bauer et al., the regular use of aspirin had a modest beneficial effect on BMD in postmenopausal women, but increased BMD was not associated with a protective effect on the risk of fractures [10]. Laura et al. also showed that the chronic use of aspirin resulted in significantly higher BMD at multiple skeletal sites in men and women [28]. However, another study demonstrated that there was no difference in BMD between chronic low-dose aspirin (≤ 125 mg) users and non-users in the general population [13]. Although the effects of aspirin are still controversial and the detailed functional mechanisms have not been completely elucidated, aspirin may exert positive biological effects on bone remodeling. In this study, we comprehensively investigated the role of aspirin in bone metabolism and the mechanism of action by which aspirin may affect bone cells, especially in a dose-dependent manner.

Excessive osteoclast activity and impaired osteoblast function are the primary characteristics of osteoporosis. Previous studies have demonstrated that aspirin generally has a bone-protective effect and prevents bone loss in animals [29]. However, the dose of aspirin used in previous studies varied greatly. In Wu’s study [30], aspirin (150 μg/ml) inhibited osteoclasts differentiation in the bone defect model. Yamaza et al. [15] demonstrated that aspirin (0.6 mg/ml) could inhibit osteoclast activity in OVX mice, leading to ameliorating bone density. In the present study, aspirin inhibited RANKL-induced osteoclastogenesis in a dose-dependent manner. Aspirin at a low dose of 10 μg/ml significantly suppressed osteoclast formation and bone resorption in vitro. This inhibitory effect was more pronounced with increasing aspirin concentrations. At a high dose of 200 μg/ml, aspirin almost completely inhibited the bone resorption function of osteoclasts. In this in vivo study, the low dose of aspirin (6 mg/kg/day), which were equivalent to the preventive dose (100 mg/day) for cardiovascular events in clinical practice, and the high dose of aspirin (30 mg/kg/day) were consecutively administered to OVX rats for 3 months. Our data showed that low-dose aspirin prevented bone mass loss induced by OVX. High-dose aspirin had a more prominent protective effect. Moreover, aspirin treatment markedly decreased the number of TRAP-positive cells in the trabecular bones of OVX rats.

The activation of NF-*κ*B signaling is the dominant mediator of osteoclast survival, differentiation, and bone resorption [31]. I*κ*B*α* sequesters NF-*κ*B subunits in the cytoplasm. The binding of RANKL to RANK leads to the phosphorylation and degradation of I*κ*B*α* and the subsequent release of the NF-*κ*B p65 subunit. The p65 subunit of NF-*κ*B translocates into the cell nucleus and binds to target sites, initiating the expression of osteoclast-specific genes. Our results demonstrated that aspirin attenuated I*κ*B*α* degradation and suppressed the activation and nuclear translocation of the NF-*κ*B p65 subunit.

The spatiotemporal role of aspirin in osteogenesis remains controversial based on previous studies. According to a study by Yamaza et al., aspirin treatment (2.5 and 50 μg/ml) facilitated the mineralized accumulation of BMMSCs in vitro [15]. Moreover, low-dose aspirin treatment (10 and 50 μg/ml) improved the osteogenic differentiation of stem cells from exfoliated deciduous teeth, whereas this boosting effect was absent when the aspirin concentration was increased to 200 μg/ml [32]. Furthermore, aspirin treatment (50, 75, 100, 150, and 200 μg/ml) stimulated osteogenesis of BMSCs, and aspirin at a concentration of 75 μg/ml showed the highest osteogenic capacity [33]. In contrast, Guida et al. demonstrated that aspirin treatment (50, 100, and 200 μg/ml) inhibits BMSCs proliferation and osteogenic differentiation in a dose-dependent manner. Aspirin at a dose of 200 μg/ml caused an extensive reduction (> 90%) in calcified deposits [34]. In our study, the results showed that low concentrations of aspirin (10, 50, and 100 μg/ml) gradually promoted osteogenic differentiation, while these facilitating effects were attenuated by high concentrations of aspirin (150, and 200 μg/ml). At a concentration of 100 μg/ml, aspirin demonstrated the maximal osteogenic differentiation capacity.

Our study had several limitations. First, aspirin irreversibly inhibits COX-1 and COX-2 enzymes. Inhibition of COX-1 prevents PGH2 formation and subsequent thromboxane A2 (TXA2). COX-2 suppression inhibits the conversion of arachidonic acid to prostaglandin E2 (PGE2). Cyclooxygenase and prostaglandins (PGs) are multifunctional coordinators of bone metabolism that regulate bone resorption and formation [35]. The functional mechanism of aspirin regulating bone metabolism through cyclooxygenase and PGs mediators needs further investigation. Second, the side effects of aspirin therapy are dose-dependent, with increased major bleeding events at high doses. However, the side effects of high-dose aspirin were not assessed in our study.

In conclusion, we demonstrated that aspirin prevents bone loss in a dual regulatory manner. Low-dose aspirin exerted beneficial effects on the preservation of bone mass by synchronously inhibiting osteoclast differentiation and promoting osteogenesis. However, high-dose aspirin prevented bone loss due to the suppression of osteoclast activity, even more so than osteogenic differentiation. Our data indicate that aspirin may have potential applications in the prevention and treatment of osteopenia.

## Data Availability

The data used to support the findings of this study are available from the corresponding author upon request.

## Abbreviations

M-CSF: Macrophage colony-stimulating factor
RANK: Receptor activator of nuclear factor-(*κ*B)
ASA: Acetylsalicylic acid
BMD: Bone mineral density
OVX: Ovariectomized
TRAP: Tartrate-resistant acid phosphatase
ALP: Alkaline phosphatase
ARS: Alizarin red S
BMMs: Bone marrow-derived macrophages
BV/TV: Bone volume/total tissue volume
Tb.N: Trabecular number
Tb.Th: Trabecular thickness
Tb.Sp: Trabecular space
CTSK: Cathepsin K
CTR: Calcitonin receptor
NFATc1: Nuclear factor of activated T-cells cytoplasmic 1
MMP9: Matrix metalloproteinase-9
